# On the visualization of universal degeneracy in the IMRT problem

**DOI:** 10.1186/1748-717X-1-47

**Published:** 2006-12-18

**Authors:** Markus L Alber, Gustav Meedt

**Affiliations:** 1 Section for Biomedical Physics, Clinic for Radiooncology, University of Tübingen, Germany; 2 Computerized Medical Systems, St. Louis, USA

## Abstract

**Background:**

In general, the IMRT optimisation problem possesses many equivalent solutions. This makes it difficult to decide whether a result produced by an IMRT planning algorithm can be further improved, e.g. by adding more beams, or whether it is close to the globally best solution.

**Results:**

It is conjectured that the curvature properties of the objective function around any globally optimum dose distribution are universal. This allows an assessment of optimality of dose distributions that are generated by different beam arrangements in a complementary manner to the objective function value alone. A tool to visualize the curvature structure of the objective function is devised.

**Conclusion:**

In an example case, it is demonstrated how the assessment of the curvature space can indicate the equivalence of rival beam configurations and their proximity to the global optimum.

## Background

Because of the dependence on optimisation algorithms, treatment planning for intensity modulated photon radiotherapy (IMRT) frequently evades intuition. This is particularly true for the issue of beam placement in complex cases that may even require non-coplanar beam directions, where it is hard to decide whether both the number and the directions of beams are optimum in the sense that the resulting dose distribution cannot be improved further with reasonable effort. The purpose of this note is to give an intuitive picture and a tool for visualization of a central property of the IMRT optimisation problem, which is the degeneracy of the solution space.

It is a practical wisdom of the field, that there exists a very large number of virtually equivalent solutions for an IMRT optimisation problem. Due to this degeneracy of the solution space, it is not necessary to find one solitary best set of beam directions and fluence weight profiles, but merely a set that is equivalent (i.e. in practice: close enough) to the global optimum. It is fair to say that the true optimum of an IMRT problem is virtually never sought: usually, a few dozen iterations of a gradient algorithm are deemed sufficient, while a non-linear optimisation problem of a few thousand parameters would typically require thousands of iterations. Eventually, it is degeneracy that makes beamlet-based IMRT optimisation tractable.

The degeneracy of the objective function is inherent to the problem and does not arise spuriously from any particular mathematical formulation. In a previous paper [1], a conjecture was made about the universality of the curvature properties in optimisation problems that differed in the number and arrangement of beams. It is helpful to imagine the situation in 2D as a long, narrow valley that is absolutely flat along its floor, but rises sharply perpendicular to it. The steep slopes on either side correspond to the high costs for increasing the dose, imposed by some normal tissue objective, and for decreasing dose, imposed by some target objective. The minimum in this direction is a finely tuned balance between these objectives. Since these objectives oppose each other for all reasonable beam arrangements, it can be assumed that the balance is sought in a very similar way for all of them. This means, that the solution space for all beam arrangements should show the same pattern of directions of great curvature, which is characteristic for the given case. It would be intriguing if this universal curvature pattern could be visualized.

In order to compare the curvature properties of the optimum dose distributions of different beam arrangements, the matrix of second derivatives for all beamlets of the union of the two beam arrangements would have to be computed. This can quickly become an onerous task. In this manuscript, we try to overcome this obstacle by introducing an independent set of arbitrary, but well chosen *probing fluence elements *which can be shared between different beam arrangements and are solely used for visualization. These probing fluence elements define a *curvature map*. In case the objective function of the optimisation problem is convex, it can be shown that all globally optimum solutions share the same subspace of zero curvature. Further, it is conjectured that the subspace of non-vanishing curvature is also an invariant. An example of this curvature map and its application in the context of manual and computerized beam direction optimisation is given for a head and neck tumour case.

## Methods

The notation of this development is introduced as follows. Let *F*(*D*) be a twice continuously differentiable objective function of the dose distribution *D *= (*D*_*j*_)_*j *= 1..*m *_in the patient volume *V *consisting of *m *voxels, whose minimum defines the desired solution. Further, let ∂2F(D)∂Di∂Dj=0
 MathType@MTEF@5@5@+=feaafiart1ev1aaatCvAUfKttLearuWrP9MDH5MBPbIqV92AaeXatLxBI9gBaebbnrfifHhDYfgasaacH8akY=wiFfYdH8Gipec8Eeeu0xXdbba9frFj0=OqFfea0dXdd9vqai=hGuQ8kuc9pgc9s8qqaq=dirpe0xb9q8qiLsFr0=vr0=vr0dc8meaabaqaciaacaGaaeqabaqabeGadaaakeaadaWcaaqaaiabgkGi2oaaCaaaleqabaGaeGOmaidaaOGaemOrayKaeiikaGIaemiraqKaeiykaKcabaGaeyOaIyRaemiraq0aaSbaaSqaaiabdMgaPbqabaGccqGHciITcqWGebardaWgaaWcbaGaemOAaOgabeaaaaGccqGH9aqpcqaIWaamaaa@3D29@ for *i *≠ *j*. All the treatment goals with respect to target coverage, target dose homogeneity and normal tissue sparing are assumed to be incorporated in this objective function. For simplicity, let *F *be a sum of local objective function densities *f*(*D*_*j*_), so that F=∑j=1mf(Dj)
 MathType@MTEF@5@5@+=feaafiart1ev1aaatCvAUfKttLearuWrP9MDH5MBPbIqV92AaeXatLxBI9gBaebbnrfifHhDYfgasaacH8akY=wiFfYdH8Gipec8Eeeu0xXdbba9frFj0=OqFfea0dXdd9vqai=hGuQ8kuc9pgc9s8qqaq=dirpe0xb9q8qiLsFr0=vr0=vr0dc8meaabaqaciaacaGaaeqabaqabeGadaaakeaacqWGgbGrcqGH9aqpdaaeWaqaaiabdAgaMjabcIcaOiabdseaenaaBaaaleaacqWGQbGAaeqaaOGaeiykaKcaleaacqWGQbGAcqGH9aqpcqaIXaqmaeaacqWGTbqBa0GaeyyeIuoaaaa@3B2A@. This does not restrict generality unduly, since most objective functions proposed for IMRT can be written in this form and fulfill the condition on the second derivative. For a proof see Romeijn et al. [2]. For example, the classical one- and two-sided quadratic penalty functions *f*(*D*) = (*D *- *D*_0_)^2 ^Θ (*D*, *D*_0_), where Θ (*D*, *D*_0_) determines whether the penalty applies to doses greater and/or smaller than a threshold *D*_0_, are of this shape (Here, it is convenient to postulate that Θ is not a step function, but a steep yet differentiable sigmoid so as to ensure the existence of a second derivative). Also, biological indices based on sums of local dose-response functions like EUD (*f *= Djk
 MathType@MTEF@5@5@+=feaafiart1ev1aaatCvAUfKttLearuWrP9MDH5MBPbIqV92AaeXatLxBI9gBaebbnrfifHhDYfgasaacH8akY=wiFfYdH8Gipec8Eeeu0xXdbba9frFj0=OqFfea0dXdd9vqai=hGuQ8kuc9pgc9s8qqaq=dirpe0xb9q8qiLsFr0=vr0=vr0dc8meaabaqaciaacaGaaeqabaqabeGadaaakeaacqWGebardaqhaaWcbaGaemOAaOgabaGaem4AaSgaaaaa@30A6@, *k *> 0), TCP (*f *= exp(-*αD*_*j*_)) and NTCP (e.g. *f *= Djk
 MathType@MTEF@5@5@+=feaafiart1ev1aaatCvAUfKttLearuWrP9MDH5MBPbIqV92AaeXatLxBI9gBaebbnrfifHhDYfgasaacH8akY=wiFfYdH8Gipec8Eeeu0xXdbba9frFj0=OqFfea0dXdd9vqai=hGuQ8kuc9pgc9s8qqaq=dirpe0xb9q8qiLsFr0=vr0=vr0dc8meaabaqaciaacaGaaeqabaqabeGadaaakeaacqWGebardaqhaaWcbaGaemOAaOgabaGaem4AaSgaaaaa@30A6@, *k *≥ 1), as well as DVH penalties (e.g. *f *= 1/(1 + (*D*_0_/*D*)^*k*^), *k *> 1) can be transformed into this 'standard form' without loss of generality [3]. The dose distribution is generated by a fluence distribution, which is by definition composed of a weighted sum of elementary fluence distributions. Let ℬ
 MathType@MTEF@5@5@+=feaafiart1ev1aaatCvAUfKttLearuWrP9MDH5MBPbIqV92AaeXatLxBI9gBamrtHrhAL1wy0L2yHvtyaeHbnfgDOvwBHrxAJfwnaebbnrfifHhDYfgasaacH8akY=wiFfYdH8Gipec8Eeeu0xXdbba9frFj0=OqFfea0dXdd9vqai=hGuQ8kuc9pgc9s8qqaq=dirpe0xb9q8qiLsFr0=vr0=vr0dc8meaabaqaciaacaGaaeqabaWaaeGaeaaakeaaimaacqWFSeIqaaa@377E@ be a basis set of elementary fluence distributions (fluence elements) (*η*_*i*_)_*i *= 1..*n*_, and (*φ*_*i*_)_*i *= 1..*n *_≤ 0 the associated weights (A fluence element can, but need not be a beamlet in the common understanding as a constituent of an intensity modulated field. A beamlet is an elementary fluence distribution with the property that a finite superposition yields a deliverable intensity modulated field). For example, ℬ
 MathType@MTEF@5@5@+=feaafiart1ev1aaatCvAUfKttLearuWrP9MDH5MBPbIqV92AaeXatLxBI9gBamrtHrhAL1wy0L2yHvtyaeHbnfgDOvwBHrxAJfwnaebbnrfifHhDYfgasaacH8akY=wiFfYdH8Gipec8Eeeu0xXdbba9frFj0=OqFfea0dXdd9vqai=hGuQ8kuc9pgc9s8qqaq=dirpe0xb9q8qiLsFr0=vr0=vr0dc8meaabaqaciaacaGaaeqabaWaaeGaeaaakeaaimaacqWFSeIqaaa@377E@ can be the set of all beamlets of an arrangement of intensity modulated beams, or the set of all arclets of radiosurgery arcs. The basis set defines the parameter space of the optimisation as the space of the weights that are associated with the fluence elements. By means of the basis ℬ
 MathType@MTEF@5@5@+=feaafiart1ev1aaatCvAUfKttLearuWrP9MDH5MBPbIqV92AaeXatLxBI9gBamrtHrhAL1wy0L2yHvtyaeHbnfgDOvwBHrxAJfwnaebbnrfifHhDYfgasaacH8akY=wiFfYdH8Gipec8Eeeu0xXdbba9frFj0=OqFfea0dXdd9vqai=hGuQ8kuc9pgc9s8qqaq=dirpe0xb9q8qiLsFr0=vr0=vr0dc8meaabaqaciaacaGaaeqabaWaaeGaeaaakeaaimaacqWFSeIqaaa@377E@, a subset of the abstract space of all physically feasible fluence distributions is mapped onto ℝ0|ℬ|
 MathType@MTEF@5@5@+=feaafiart1ev1aaatCvAUfKttLearuWrP9MDH5MBPbIqV92AaeXatLxBI9gBamrtHrhAL1wy0L2yHvtyaeHbnfgDOvwBHrxAJfwnaebbnrfifHhDYfgasaacH8akY=wiFfYdH8Gipec8Eeeu0xXdbba9frFj0=OqFfea0dXdd9vqai=hGuQ8kuc9pgc9s8qqaq=dirpe0xb9q8qiLsFr0=vr0=vr0dc8meaabaqaciaacaGaaeqabaWaaeGaeaaakeaat0uy0HwzTfgDPnwy3aqeh0uy0HwzTfgDPnwy3aacfaGae8xhHi1aa0baaSqaaiabicdaWaqaaiabcYha8HWaaiab+XsicjabcYha8baaaaa@460F@. This is in analogy to geometry, where e.g. a point in space can be identified by its coordinates relative to a set of basis vectors.

Let (*T*_*j*_)_*j *= 1..*m *_be the (linear) dose deposition operator that associates the fluence element *η*_*i *_with its dose distribution (*T*_*j*_)_*j *= 1..*m *_*η*_*i *_= (*T*_*ji*_)_*j *= 1..*m*_. Hence,

Dj=∑i=1nTjiφi.     (1)
 MathType@MTEF@5@5@+=feaafiart1ev1aaatCvAUfKttLearuWrP9MDH5MBPbIqV92AaeXatLxBI9gBaebbnrfifHhDYfgasaacH8akY=wiFfYdH8Gipec8Eeeu0xXdbba9frFj0=OqFfea0dXdd9vqai=hGuQ8kuc9pgc9s8qqaq=dirpe0xb9q8qiLsFr0=vr0=vr0dc8meaabaqaciaacaGaaeqabaqabeGadaaakeaacqWGebardaWgaaWcbaGaemOAaOgabeaakiabg2da9maaqahabaGaemivaq1aaSbaaSqaaiabdQgaQjabdMgaPbqabaaabaGaemyAaKMaeyypa0JaeGymaedabaGaemOBa4ganiabggHiLdacciGccqWFgpGzdaWgaaWcbaGaemyAaKgabeaakiabc6caUiaaxMaacaWLjaWaaeWaaeaacqaIXaqmaiaawIcacaGLPaaaaaa@4354@

The solution of the IMRT problem is a vector of fluence weights (φi∗
 MathType@MTEF@5@5@+=feaafiart1ev1aaatCvAUfKttLearuWrP9MDH5MBPbIqV92AaeXatLxBI9gBaebbnrfifHhDYfgasaacH8akY=wiFfYdH8Gipec8Eeeu0xXdbba9frFj0=OqFfea0dXdd9vqai=hGuQ8kuc9pgc9s8qqaq=dirpe0xb9q8qiLsFr0=vr0=vr0dc8meaabaqaciaacaGaaeqabaqabeGadaaakeaaiiGacqWFgpGzdaqhaaWcbaGaemyAaKgabaGaey4fIOcaaaaa@30E3@)_*i *= 1..*n *_which obtains from the minimization of *F *under the constraints *φ*_*i*_≥ 0. Given that *F *is assumed to be twice continuously differentiable, the necessary optimality conditions

∂F(D∗)∂φi=0orφi∗=0∀ηi∈ℬ     (2)
 MathType@MTEF@5@5@+=feaafiart1ev1aaatCvAUfKttLearuWrP9MDH5MBPbIqV92AaeXatLxBI9gBaebbnrfifHhDYfgasaacH8akY=wiFfYdH8Gipec8Eeeu0xXdbba9frFj0=OqFfea0dXdd9vqai=hGuQ8kuc9pgc9s8qqaq=dirpe0xb9q8qiLsFr0=vr0=vr0dc8meaabaqaciaacaGaaeqabaqabeGadaaakeaafaqabeqafaaaaeaadaWcaaqaaiabgkGi2kabdAeagjabcIcaOiabdseaenaaCaaaleqabaGaey4fIOcaaOGaeiykaKcabaGaeyOaIylcciGae8NXdy2aaSbaaSqaaiabdMgaPbqabaaaaOGaeyypa0JaeGimaadabaGaee4Ba8MaeeOCaihabaGae8NXdy2aa0baaSqaaiabdMgaPbqaaiabgEHiQaaakiabg2da9iabicdaWaqaaiabgcGiIaqaaiab=D7aOnaaBaaaleaacqWGPbqAaeqaaOGaeyicI4maamrtHrhAL1wy0L2yHvtyaeHbnfgDOvwBHrxAJfwnaGabaiab+XsicjaaxMaacaWLjaWaaeWaaeaacqaIYaGmaiaawIcacaGLPaaaaaa@56F2@

hold. If all *f *are convex, *F *is also convex, and by virtue of the convexity of the feasible set ℱ
 MathType@MTEF@5@5@+=feaafiart1ev1aaatCvAUfKttLearuWrP9MDH5MBPbIqV92AaeXatLxBI9gBamrtHrhAL1wy0L2yHvtyaeHbnfgDOvwBHrxAJfwnaebbnrfifHhDYfgasaacH8akY=wiFfYdH8Gipec8Eeeu0xXdbba9frFj0=OqFfea0dXdd9vqai=hGuQ8kuc9pgc9s8qqaq=dirpe0xb9q8qiLsFr0=vr0=vr0dc8meaabaqaciaacaGaaeqabaWaaeGaeaaakeaaimaacqWFXeIraaa@3787@ = {*φ*_*i*_∈ℝ|ℬ|
 MathType@MTEF@5@5@+=feaafiart1ev1aaatCvAUfKttLearuWrP9MDH5MBPbIqV92AaeXatLxBI9gBamrtHrhAL1wy0L2yHvtyaeHbnfgDOvwBHrxAJfwnaebbnrfifHhDYfgasaacH8akY=wiFfYdH8Gipec8Eeeu0xXdbba9frFj0=OqFfea0dXdd9vqai=hGuQ8kuc9pgc9s8qqaq=dirpe0xb9q8qiLsFr0=vr0=vr0dc8meaabaqaciaacaGaaeqabaWaaeGaeaaakeaat0uy0HwzTfgDPnwy3aqeh0uy0HwzTfgDPnwy3aacfaGae8xhHi1aaWbaaSqabeaaieaacqGF8baFimaacqqFSeIqcqGF8baFaaaaaa@4521@ : *φ*_*i *_≥ 0}, only a single global minimum exists (generally, the dose limiting objectives ensure that *F *is bounded from below and *F *→ ∞ for all *φ*_*i *_→ ∞). However, this does *not *mean that the minimum is attained in a single point. In the case of degeneracy, as commonly present in the IMRT setting, the *set of minimizers *is a sizeable, high dimensional, closed and connected subset of the parameter space. Any such point in this solution space is indistinguishable from any other with respect to its objective function value. The uniqueness of the minimizer cannot be guaranteed even if the objective densities *f *are strictly convex, because *F*(*φ*) is a composition of monotonously increasing and decreasing convex functions. In practice, this causes IMRT optimisation algorithms to terminate at different solutions if started from slightly different points, which may be mistaken for local, isolated minima. The situation is different in case either *f *or ℱ
 MathType@MTEF@5@5@+=feaafiart1ev1aaatCvAUfKttLearuWrP9MDH5MBPbIqV92AaeXatLxBI9gBamrtHrhAL1wy0L2yHvtyaeHbnfgDOvwBHrxAJfwnaebbnrfifHhDYfgasaacH8akY=wiFfYdH8Gipec8Eeeu0xXdbba9frFj0=OqFfea0dXdd9vqai=hGuQ8kuc9pgc9s8qqaq=dirpe0xb9q8qiLsFr0=vr0=vr0dc8meaabaqaciaacaGaaeqabaWaaeGaeaaakeaaimaacqWFXeIraaa@3787@ are non-convex, which can occur when dose-volume objectives are employed or MLC constraints need to be considered. In this case the multiple global minimizers can lie in disconnected and non-convex sets, or local minima may exist. Notice that these are possibilities, not necessities.

If two different dose distributions, possibly generated by different beam arrangements, have the same objective function value, they may be degenerate to the global solution, or merely be local solutions that share the same objective function value by coincidence. By virtue of the linearity of the dose operator *T*, there exists an easy test to investigate this situation. Let ℬ
 MathType@MTEF@5@5@+=feaafiart1ev1aaatCvAUfKttLearuWrP9MDH5MBPbIqV92AaeXatLxBI9gBamrtHrhAL1wy0L2yHvtyaeHbnfgDOvwBHrxAJfwnaebbnrfifHhDYfgasaacH8akY=wiFfYdH8Gipec8Eeeu0xXdbba9frFj0=OqFfea0dXdd9vqai=hGuQ8kuc9pgc9s8qqaq=dirpe0xb9q8qiLsFr0=vr0=vr0dc8meaabaqaciaacaGaaeqabaWaaeGaeaaakeaaimaacqWFSeIqaaa@377E@_1_, ℬ
 MathType@MTEF@5@5@+=feaafiart1ev1aaatCvAUfKttLearuWrP9MDH5MBPbIqV92AaeXatLxBI9gBamrtHrhAL1wy0L2yHvtyaeHbnfgDOvwBHrxAJfwnaebbnrfifHhDYfgasaacH8akY=wiFfYdH8Gipec8Eeeu0xXdbba9frFj0=OqFfea0dXdd9vqai=hGuQ8kuc9pgc9s8qqaq=dirpe0xb9q8qiLsFr0=vr0=vr0dc8meaabaqaciaacaGaaeqabaWaaeGaeaaakeaaimaacqWFSeIqaaa@377E@_2 _be two fluence basis sets, for example the sets of all beamlets of two arrangements of beam directions, and D1∗
 MathType@MTEF@5@5@+=feaafiart1ev1aaatCvAUfKttLearuWrP9MDH5MBPbIqV92AaeXatLxBI9gBaebbnrfifHhDYfgasaacH8akY=wiFfYdH8Gipec8Eeeu0xXdbba9frFj0=OqFfea0dXdd9vqai=hGuQ8kuc9pgc9s8qqaq=dirpe0xb9q8qiLsFr0=vr0=vr0dc8meaabaqaciaacaGaaeqabaqabeGadaaakeaacqWGebardaqhaaWcbaGaeGymaedabaGaey4fIOcaaaaa@2FC9@, D2∗
 MathType@MTEF@5@5@+=feaafiart1ev1aaatCvAUfKttLearuWrP9MDH5MBPbIqV92AaeXatLxBI9gBaebbnrfifHhDYfgasaacH8akY=wiFfYdH8Gipec8Eeeu0xXdbba9frFj0=OqFfea0dXdd9vqai=hGuQ8kuc9pgc9s8qqaq=dirpe0xb9q8qiLsFr0=vr0=vr0dc8meaabaqaciaacaGaaeqabaqabeGadaaakeaacqWGebardaqhaaWcbaGaeGOmaidabaGaey4fIOcaaaaa@2FCB@ be the associated optimum dose distributions. Further, let *F*(D1∗
 MathType@MTEF@5@5@+=feaafiart1ev1aaatCvAUfKttLearuWrP9MDH5MBPbIqV92AaeXatLxBI9gBaebbnrfifHhDYfgasaacH8akY=wiFfYdH8Gipec8Eeeu0xXdbba9frFj0=OqFfea0dXdd9vqai=hGuQ8kuc9pgc9s8qqaq=dirpe0xb9q8qiLsFr0=vr0=vr0dc8meaabaqaciaacaGaaeqabaqabeGadaaakeaacqWGebardaqhaaWcbaGaeGymaedabaGaey4fIOcaaaaa@2FC9@) = *F*(D2∗
 MathType@MTEF@5@5@+=feaafiart1ev1aaatCvAUfKttLearuWrP9MDH5MBPbIqV92AaeXatLxBI9gBaebbnrfifHhDYfgasaacH8akY=wiFfYdH8Gipec8Eeeu0xXdbba9frFj0=OqFfea0dXdd9vqai=hGuQ8kuc9pgc9s8qqaq=dirpe0xb9q8qiLsFr0=vr0=vr0dc8meaabaqaciaacaGaaeqabaqabeGadaaakeaacqWGebardaqhaaWcbaGaeGOmaidabaGaey4fIOcaaaaa@2FCB@). For 0 ≤ *λ *≤ 1, we define

*F*(*λ*) = *F*(*λ *D1∗
 MathType@MTEF@5@5@+=feaafiart1ev1aaatCvAUfKttLearuWrP9MDH5MBPbIqV92AaeXatLxBI9gBaebbnrfifHhDYfgasaacH8akY=wiFfYdH8Gipec8Eeeu0xXdbba9frFj0=OqFfea0dXdd9vqai=hGuQ8kuc9pgc9s8qqaq=dirpe0xb9q8qiLsFr0=vr0=vr0dc8meaabaqaciaacaGaaeqabaqabeGadaaakeaacqWGebardaqhaaWcbaGaeGymaedabaGaey4fIOcaaaaa@2FC9@ + (1 - *λ*)D2∗
 MathType@MTEF@5@5@+=feaafiart1ev1aaatCvAUfKttLearuWrP9MDH5MBPbIqV92AaeXatLxBI9gBaebbnrfifHhDYfgasaacH8akY=wiFfYdH8Gipec8Eeeu0xXdbba9frFj0=OqFfea0dXdd9vqai=hGuQ8kuc9pgc9s8qqaq=dirpe0xb9q8qiLsFr0=vr0=vr0dc8meaabaqaciaacaGaaeqabaqabeGadaaakeaacqWGebardaqhaaWcbaGaeGOmaidabaGaey4fIOcaaaaa@2FCB@)     (3)

and compute *F*(*λ*) for a small number of *λ *∈ [0, 1]. This test does not entail more than the weighted addition of two dose distributions and repeated evaluations of the objective function, but no operations in fluence weight space.

If there exists one *λ' *with *F*(*λ'*) >*F*(D1∗
 MathType@MTEF@5@5@+=feaafiart1ev1aaatCvAUfKttLearuWrP9MDH5MBPbIqV92AaeXatLxBI9gBaebbnrfifHhDYfgasaacH8akY=wiFfYdH8Gipec8Eeeu0xXdbba9frFj0=OqFfea0dXdd9vqai=hGuQ8kuc9pgc9s8qqaq=dirpe0xb9q8qiLsFr0=vr0=vr0dc8meaabaqaciaacaGaaeqabaqabeGadaaakeaacqWGebardaqhaaWcbaGaeGymaedabaGaey4fIOcaaaaa@2FC9@), then obviously both dose distributions do not belong to the same minimum. This situation can only occur if *F *is non-convex. If *F *is convex, and D1∗
 MathType@MTEF@5@5@+=feaafiart1ev1aaatCvAUfKttLearuWrP9MDH5MBPbIqV92AaeXatLxBI9gBaebbnrfifHhDYfgasaacH8akY=wiFfYdH8Gipec8Eeeu0xXdbba9frFj0=OqFfea0dXdd9vqai=hGuQ8kuc9pgc9s8qqaq=dirpe0xb9q8qiLsFr0=vr0=vr0dc8meaabaqaciaacaGaaeqabaqabeGadaaakeaacqWGebardaqhaaWcbaGaeGymaedabaGaey4fIOcaaaaa@2FC9@, D2∗
 MathType@MTEF@5@5@+=feaafiart1ev1aaatCvAUfKttLearuWrP9MDH5MBPbIqV92AaeXatLxBI9gBaebbnrfifHhDYfgasaacH8akY=wiFfYdH8Gipec8Eeeu0xXdbba9frFj0=OqFfea0dXdd9vqai=hGuQ8kuc9pgc9s8qqaq=dirpe0xb9q8qiLsFr0=vr0=vr0dc8meaabaqaciaacaGaaeqabaqabeGadaaakeaacqWGebardaqhaaWcbaGaeGOmaidabaGaey4fIOcaaaaa@2FCB@ are degenerate to the global minimum, then

*F*(D1∗
 MathType@MTEF@5@5@+=feaafiart1ev1aaatCvAUfKttLearuWrP9MDH5MBPbIqV92AaeXatLxBI9gBaebbnrfifHhDYfgasaacH8akY=wiFfYdH8Gipec8Eeeu0xXdbba9frFj0=OqFfea0dXdd9vqai=hGuQ8kuc9pgc9s8qqaq=dirpe0xb9q8qiLsFr0=vr0=vr0dc8meaabaqaciaacaGaaeqabaqabeGadaaakeaacqWGebardaqhaaWcbaGaeGymaedabaGaey4fIOcaaaaa@2FC9@) = *F*(D*) = *F*(*λ*D1∗
 MathType@MTEF@5@5@+=feaafiart1ev1aaatCvAUfKttLearuWrP9MDH5MBPbIqV92AaeXatLxBI9gBaebbnrfifHhDYfgasaacH8akY=wiFfYdH8Gipec8Eeeu0xXdbba9frFj0=OqFfea0dXdd9vqai=hGuQ8kuc9pgc9s8qqaq=dirpe0xb9q8qiLsFr0=vr0=vr0dc8meaabaqaciaacaGaaeqabaqabeGadaaakeaacqWGebardaqhaaWcbaGaeGymaedabaGaey4fIOcaaaaa@2FC9@ + (1 - *λ*)D*).     (4)

By definition, *F *is always locally convex in a certain environment of the global minimum (otherwise it would not be a minimum), which makes the following argumentation applicable to some extent also to the globally non-convex case. In the following, we use eq. 4 to motivate a conjecture about universal curvature properties of all solutions that are degenerate to the global optimum. In all but the most contrived cases, the comprehensive basis ℬ
 MathType@MTEF@5@5@+=feaafiart1ev1aaatCvAUfKttLearuWrP9MDH5MBPbIqV92AaeXatLxBI9gBamrtHrhAL1wy0L2yHvtyaeHbnfgDOvwBHrxAJfwnaebbnrfifHhDYfgasaacH8akY=wiFfYdH8Gipec8Eeeu0xXdbba9frFj0=OqFfea0dXdd9vqai=hGuQ8kuc9pgc9s8qqaq=dirpe0xb9q8qiLsFr0=vr0=vr0dc8meaabaqaciaacaGaaeqabaWaaeGaeaaakeaaimaacqWFSeIqaaa@377E@* of all globally optimum dose distributions, i.e. the union of all basis sets that generate a global optimum, is very large. Simultaneously, a very large number of degenerate and near-degenerate solutions exist. Assume that some dose distribution D1∗
 MathType@MTEF@5@5@+=feaafiart1ev1aaatCvAUfKttLearuWrP9MDH5MBPbIqV92AaeXatLxBI9gBaebbnrfifHhDYfgasaacH8akY=wiFfYdH8Gipec8Eeeu0xXdbba9frFj0=OqFfea0dXdd9vqai=hGuQ8kuc9pgc9s8qqaq=dirpe0xb9q8qiLsFr0=vr0=vr0dc8meaabaqaciaacaGaaeqabaqabeGadaaakeaacqWGebardaqhaaWcbaGaeGymaedabaGaey4fIOcaaaaa@2FC9@ is degenerate to the global optimum D*, i.e. *F*(D1∗
 MathType@MTEF@5@5@+=feaafiart1ev1aaatCvAUfKttLearuWrP9MDH5MBPbIqV92AaeXatLxBI9gBaebbnrfifHhDYfgasaacH8akY=wiFfYdH8Gipec8Eeeu0xXdbba9frFj0=OqFfea0dXdd9vqai=hGuQ8kuc9pgc9s8qqaq=dirpe0xb9q8qiLsFr0=vr0=vr0dc8meaabaqaciaacaGaaeqabaqabeGadaaakeaacqWGebardaqhaaWcbaGaeGymaedabaGaey4fIOcaaaaa@2FC9@) = *F*(D*). By the above definition, ℬ
 MathType@MTEF@5@5@+=feaafiart1ev1aaatCvAUfKttLearuWrP9MDH5MBPbIqV92AaeXatLxBI9gBamrtHrhAL1wy0L2yHvtyaeHbnfgDOvwBHrxAJfwnaebbnrfifHhDYfgasaacH8akY=wiFfYdH8Gipec8Eeeu0xXdbba9frFj0=OqFfea0dXdd9vqai=hGuQ8kuc9pgc9s8qqaq=dirpe0xb9q8qiLsFr0=vr0=vr0dc8meaabaqaciaacaGaaeqabaWaaeGaeaaakeaaimaacqWFSeIqaaa@377E@_1 _⊂ ℬ
 MathType@MTEF@5@5@+=feaafiart1ev1aaatCvAUfKttLearuWrP9MDH5MBPbIqV92AaeXatLxBI9gBamrtHrhAL1wy0L2yHvtyaeHbnfgDOvwBHrxAJfwnaebbnrfifHhDYfgasaacH8akY=wiFfYdH8Gipec8Eeeu0xXdbba9frFj0=OqFfea0dXdd9vqai=hGuQ8kuc9pgc9s8qqaq=dirpe0xb9q8qiLsFr0=vr0=vr0dc8meaabaqaciaacaGaaeqabaWaaeGaeaaakeaaimaacqWFSeIqaaa@377E@*. The optimality conditions have to be satisfied for all elements of ℬ
 MathType@MTEF@5@5@+=feaafiart1ev1aaatCvAUfKttLearuWrP9MDH5MBPbIqV92AaeXatLxBI9gBamrtHrhAL1wy0L2yHvtyaeHbnfgDOvwBHrxAJfwnaebbnrfifHhDYfgasaacH8akY=wiFfYdH8Gipec8Eeeu0xXdbba9frFj0=OqFfea0dXdd9vqai=hGuQ8kuc9pgc9s8qqaq=dirpe0xb9q8qiLsFr0=vr0=vr0dc8meaabaqaciaacaGaaeqabaWaaeGaeaaakeaaimaacqWFSeIqaaa@377E@*. We apply the optimality conditions eq. 2 to the right hand side of eq. 4

∂f(λD1∗+(1−λ)D∗)∂φi=∑j=1m∂f(λD1∗+(1−λ)D∗)∂Dj⋅Tji=0∀ηi∈ℬ∗.     (5)
 MathType@MTEF@5@5@+=feaafiart1ev1aaatCvAUfKttLearuWrP9MDH5MBPbIqV92AaeXatLxBI9gBamrtHrhAL1wy0L2yHvtyaeHbnfgDOvwBHrxAJfwnaebbnrfifHhDYfgasaacH8akY=wiFfYdH8Gipec8Eeeu0xXdbba9frFj0=OqFfea0dXdd9vqai=hGuQ8kuc9pgc9s8qqaq=dirpe0xb9q8qiLsFr0=vr0=vr0dc8meaabaqaciaacaGaaeqabaWaaeGaeaaakeaafaqadeGadaaabaWaaSaaaeaacqGHciITcqWGMbGzcqGGOaakiiGacqWF7oaBcqWGebardaqhaaWcbaGaeGymaedabaGaey4fIOcaaOGaey4kaSIaeiikaGIaeGymaeJaeyOeI0Iae83UdWMaeiykaKIaemiraq0aaWbaaSqabeaacqGHxiIkaaGccqGGPaqkaeaacqGHciITcqWFgpGzdaWgaaWcbaGaemyAaKgabeaaaaGccqGH9aqpaeaaaeaaaeaadaaeWbqaamaalaaabaGaeyOaIyRaemOzayMaeiikaGIae83UdWMaemiraq0aa0baaSqaaiabigdaXaqaaiabgEHiQaaakiabgUcaRiabcIcaOiabigdaXiabgkHiTiab=T7aSjabcMcaPiabdseaenaaCaaaleqabaGaey4fIOcaaOGaeiykaKcabaGaeyOaIyRaemiraq0aaSbaaSqaaiabdQgaQbqabaaaaaqaaiabdQgaQjabg2da9iabigdaXaqaaiabd2gaTbqdcqGHris5aOGaeyyXICTaemivaq1aaSbaaSqaaiabdQgaQjabdMgaPbqabaGccqGH9aqpcqaIWaamaeaacqGHaiIiaeaacqWF3oaAdaWgaaWcbaGaemyAaKgabeaakiabgIGioJWaaiab+XsicnaaCaaaleqabaGaey4fIOcaaOGaeiOla4caaiaaxMaacaWLjaWaaeWaaeaacqaI1aqnaiaawIcacaGLPaaaaaa@7F52@

We expand the left hand side to first order in *λ*, using that ∂2f(D)∂Di∂Dj=0
 MathType@MTEF@5@5@+=feaafiart1ev1aaatCvAUfKttLearuWrP9MDH5MBPbIqV92AaeXatLxBI9gBaebbnrfifHhDYfgasaacH8akY=wiFfYdH8Gipec8Eeeu0xXdbba9frFj0=OqFfea0dXdd9vqai=hGuQ8kuc9pgc9s8qqaq=dirpe0xb9q8qiLsFr0=vr0=vr0dc8meaabaqaciaacaGaaeqabaqabeGadaaakeaadaWcaaqaaiabgkGi2oaaCaaaleqabaGaeGOmaidaaOGaemOzayMaeiikaGIaemiraqKaeiykaKcabaGaeyOaIyRaemiraq0aaSbaaSqaaiabdMgaPbqabaGccqGHciITcqWGebardaWgaaWcbaGaemOAaOgabeaaaaGccqGH9aqpcqaIWaamaaa@3D69@

lhs=0+λ∑j=1m(D1,j∗−Dj∗)∂2f(D∗)∂Dj2Tji+O(λ2)∀ηi∈ℬ∗.     (6)
 MathType@MTEF@5@5@+=feaafiart1ev1aaatCvAUfKttLearuWrP9MDH5MBPbIqV92AaeXatLxBI9gBamrtHrhAL1wy0L2yHvtyaeHbnfgDOvwBHrxAJfwnaebbnrfifHhDYfgasaacH8akY=wiFfYdH8Gipec8Eeeu0xXdbba9frFj0=OqFfea0dXdd9vqai=hGuQ8kuc9pgc9s8qqaq=dirpe0xb9q8qiLsFr0=vr0=vr0dc8meaabaqaciaacaGaaeqabaWaaeGaeaaakeaafaqabeqadaaabaGaeeiBaWMaeeiAaGMaee4CamNaeyypa0JaeGimaaJaey4kaSccciGae83UdW2aaabCaeaacqGGOaakcqWGebardaqhaaWcbaGaeGymaeJaeiilaWIaemOAaOgabaGaey4fIOcaaOGaeyOeI0Iaemiraq0aa0baaSqaaiabdQgaQbqaaiabgEHiQaaakiabcMcaPaWcbaGaemOAaOMaeyypa0JaeGymaedabaGaemyBa0ganiabggHiLdGcdaWcaaqaaiabgkGi2oaaCaaaleqabaGaeGOmaidaaOGaemOzayMaeiikaGIaemiraq0aaWbaaSqabeaacqGHxiIkaaGccqGGPaqkaeaacqGHciITcqWGebardaqhaaWcbaGaemOAaOgabaGaeGOmaidaaaaakiabdsfaunaaBaaaleaacqWGQbGAcqWGPbqAaeqaaOGaey4kaSccdaGae4NdX=KaeiikaGIae83UdW2aaWbaaSqabeaacqaIYaGmaaGccqGGPaqkaeaacqGHaiIiaeaacqWF3oaAdaWgaaWcbaGaemyAaKgabeaakiabgIGiolab+XsicnaaCaaaleqabaGaey4fIOcaaaaakiabc6caUiaaxMaacaWLjaWaaeWaaeaacqaI2aGnaiaawIcacaGLPaaaaaa@766D@

Since D1∗
 MathType@MTEF@5@5@+=feaafiart1ev1aaatCvAUfKttLearuWrP9MDH5MBPbIqV92AaeXatLxBI9gBaebbnrfifHhDYfgasaacH8akY=wiFfYdH8Gipec8Eeeu0xXdbba9frFj0=OqFfea0dXdd9vqai=hGuQ8kuc9pgc9s8qqaq=dirpe0xb9q8qiLsFr0=vr0=vr0dc8meaabaqaciaacaGaaeqabaqabeGadaaakeaacqWGebardaqhaaWcbaGaeGymaedabaGaey4fIOcaaaaa@2FC9@ is degenerate to D*, the left hand side vanishes, i.e.

∑j=1m(D1,j∗−Dj∗)∂2f(D∗)∂Dj2Tji=0∀ηi∈ℬ∗.     (7)
 MathType@MTEF@5@5@+=feaafiart1ev1aaatCvAUfKttLearuWrP9MDH5MBPbIqV92AaeXatLxBI9gBamrtHrhAL1wy0L2yHvtyaeHbnfgDOvwBHrxAJfwnaebbnrfifHhDYfgasaacH8akY=wiFfYdH8Gipec8Eeeu0xXdbba9frFj0=OqFfea0dXdd9vqai=hGuQ8kuc9pgc9s8qqaq=dirpe0xb9q8qiLsFr0=vr0=vr0dc8meaabaqaciaacaGaaeqabaWaaeGaeaaakeaafaqabeqadaaabaWaaabCaeaacqGGOaakcqWGebardaqhaaWcbaGaeGymaeJaeiilaWIaemOAaOgabaGaey4fIOcaaOGaeyOeI0Iaemiraq0aa0baaSqaaiabdQgaQbqaaiabgEHiQaaakiabcMcaPaWcbaGaemOAaOMaeyypa0JaeGymaedabaGaemyBa0ganiabggHiLdGcdaWcaaqaaiabgkGi2oaaCaaaleqabaGaeGOmaidaaOGaemOzayMaeiikaGIaemiraq0aaWbaaSqabeaacqGHxiIkaaGccqGGPaqkaeaacqGHciITcqWGebardaqhaaWcbaGaemOAaOgabaGaeGOmaidaaaaakiabdsfaunaaBaaaleaacqWGQbGAcqWGPbqAaeqaaOGaeyypa0JaeGimaadabaGaeyiaIicabaacciGae83TdG2aaSbaaSqaaiabdMgaPbqabaGccqGHiiIZimaacqGFSeIqdaahaaWcbeqaaiabgEHiQaaaaaGccqGGUaGlcaWLjaGaaCzcamaabmaabaGaeG4naCdacaGLOaGaayzkaaaaaa@687D@

In order to interprete this condition, it is helpful to observe the Hessian matrix of second derivatives with respect to the fluence weights

Hij(D)=∑k=1mTkiT∂2f(D)∂Dk2Tkj.     (8)
 MathType@MTEF@5@5@+=feaafiart1ev1aaatCvAUfKttLearuWrP9MDH5MBPbIqV92AaeXatLxBI9gBaebbnrfifHhDYfgasaacH8akY=wiFfYdH8Gipec8Eeeu0xXdbba9frFj0=OqFfea0dXdd9vqai=hGuQ8kuc9pgc9s8qqaq=dirpe0xb9q8qiLsFr0=vr0=vr0dc8meaabaqaciaacaGaaeqabaqabeGadaaakeaacqWGibasdaWgaaWcbaGaemyAaKMaemOAaOgabeaakiabcIcaOiabdseaejabcMcaPiabg2da9maaqahabaGaemivaq1aa0baaSqaaiabdUgaRjabdMgaPbqaaiabdsfaubaaaeaacqWGRbWAcqGH9aqpcqaIXaqmaeaacqWGTbqBa0GaeyyeIuoakmaalaaabaGaeyOaIy7aaWbaaSqabeaacqaIYaGmaaGccqWGMbGzcqGGOaakcqWGebarcqGGPaqkaeaacqGHciITcqWGebardaqhaaWcbaGaem4AaSgabaGaeGOmaidaaaaakiabdsfaunaaBaaaleaacqWGRbWAcqWGQbGAaeqaaOGaeiOla4IaaCzcaiaaxMaadaqadaqaaiabiIda4aGaayjkaiaawMcaaaaa@5546@

Notice that *H*_*ij *_is a symmetric, but not diagonal matrix, while the inner second derivative of the objective function with respect to the local dose is diagonal by definition. Commonly, the vast majority of eigenvalues of this matrix is zero, owing to the high degeneracy of the IMRT problem, while the solution is characterized by the (smaller) subspace of non-vanishing curvature [1]. By comparison with eq. 7, it becomes apparent that this criterion evaluates whether the difference between two degenerate global solutions (D1∗
 MathType@MTEF@5@5@+=feaafiart1ev1aaatCvAUfKttLearuWrP9MDH5MBPbIqV92AaeXatLxBI9gBaebbnrfifHhDYfgasaacH8akY=wiFfYdH8Gipec8Eeeu0xXdbba9frFj0=OqFfea0dXdd9vqai=hGuQ8kuc9pgc9s8qqaq=dirpe0xb9q8qiLsFr0=vr0=vr0dc8meaabaqaciaacaGaaeqabaqabeGadaaakeaacqWGebardaqhaaWcbaGaeGymaedabaGaey4fIOcaaaaa@2FC9@ - D*)_*j *_= Σ_*i*_(φi∗
 MathType@MTEF@5@5@+=feaafiart1ev1aaatCvAUfKttLearuWrP9MDH5MBPbIqV92AaeXatLxBI9gBaebbnrfifHhDYfgasaacH8akY=wiFfYdH8Gipec8Eeeu0xXdbba9frFj0=OqFfea0dXdd9vqai=hGuQ8kuc9pgc9s8qqaq=dirpe0xb9q8qiLsFr0=vr0=vr0dc8meaabaqaciaacaGaaeqabaqabeGadaaakeaaiiGacqWFgpGzdaqhaaWcbaGaemyAaKgabaGaey4fIOcaaaaa@30E3@ - φ*)_*i*_*T*_*ji*_, lies entirely in the subspace of zero curvature of the global optimum. Since this has to hold for any two globally optimum dose distributions, their curvature matrices *H*_*ij*_(D*) share the same subspace of zero curvature (To see this, assume there exists a direction *η*Δ = D1∗
 MathType@MTEF@5@5@+=feaafiart1ev1aaatCvAUfKttLearuWrP9MDH5MBPbIqV92AaeXatLxBI9gBaebbnrfifHhDYfgasaacH8akY=wiFfYdH8Gipec8Eeeu0xXdbba9frFj0=OqFfea0dXdd9vqai=hGuQ8kuc9pgc9s8qqaq=dirpe0xb9q8qiLsFr0=vr0=vr0dc8meaabaqaciaacaGaaeqabaqabeGadaaakeaacqWGebardaqhaaWcbaGaeGymaedabaGaey4fIOcaaaaa@2FC9@ - D2∗
 MathType@MTEF@5@5@+=feaafiart1ev1aaatCvAUfKttLearuWrP9MDH5MBPbIqV92AaeXatLxBI9gBaebbnrfifHhDYfgasaacH8akY=wiFfYdH8Gipec8Eeeu0xXdbba9frFj0=OqFfea0dXdd9vqai=hGuQ8kuc9pgc9s8qqaq=dirpe0xb9q8qiLsFr0=vr0=vr0dc8meaabaqaciaacaGaaeqabaqabeGadaaakeaacqWGebardaqhaaWcbaGaeGOmaidabaGaey4fIOcaaaaa@2FCB@ for which the curvature around D1∗
 MathType@MTEF@5@5@+=feaafiart1ev1aaatCvAUfKttLearuWrP9MDH5MBPbIqV92AaeXatLxBI9gBaebbnrfifHhDYfgasaacH8akY=wiFfYdH8Gipec8Eeeu0xXdbba9frFj0=OqFfea0dXdd9vqai=hGuQ8kuc9pgc9s8qqaq=dirpe0xb9q8qiLsFr0=vr0=vr0dc8meaabaqaciaacaGaaeqabaqabeGadaaakeaacqWGebardaqhaaWcbaGaeGymaedabaGaey4fIOcaaaaa@2FC9@ is zero, but around D2∗
 MathType@MTEF@5@5@+=feaafiart1ev1aaatCvAUfKttLearuWrP9MDH5MBPbIqV92AaeXatLxBI9gBaebbnrfifHhDYfgasaacH8akY=wiFfYdH8Gipec8Eeeu0xXdbba9frFj0=OqFfea0dXdd9vqai=hGuQ8kuc9pgc9s8qqaq=dirpe0xb9q8qiLsFr0=vr0=vr0dc8meaabaqaciaacaGaaeqabaqabeGadaaakeaacqWGebardaqhaaWcbaGaeGOmaidabaGaey4fIOcaaaaa@2FCB@ is greater than zero. In a convex setting, it is possible to go from D1∗
 MathType@MTEF@5@5@+=feaafiart1ev1aaatCvAUfKttLearuWrP9MDH5MBPbIqV92AaeXatLxBI9gBaebbnrfifHhDYfgasaacH8akY=wiFfYdH8Gipec8Eeeu0xXdbba9frFj0=OqFfea0dXdd9vqai=hGuQ8kuc9pgc9s8qqaq=dirpe0xb9q8qiLsFr0=vr0=vr0dc8meaabaqaciaacaGaaeqabaqabeGadaaakeaacqWGebardaqhaaWcbaGaeGymaedabaGaey4fIOcaaaaa@2FC9@ to D2∗
 MathType@MTEF@5@5@+=feaafiart1ev1aaatCvAUfKttLearuWrP9MDH5MBPbIqV92AaeXatLxBI9gBaebbnrfifHhDYfgasaacH8akY=wiFfYdH8Gipec8Eeeu0xXdbba9frFj0=OqFfea0dXdd9vqai=hGuQ8kuc9pgc9s8qqaq=dirpe0xb9q8qiLsFr0=vr0=vr0dc8meaabaqaciaacaGaaeqabaqabeGadaaakeaacqWGebardaqhaaWcbaGaeGOmaidabaGaey4fIOcaaaaa@2FCB@ along a straight line without leaving the solution space, but the gradient of *F *in the direction of *η*Δ has to increase when going from D2∗
 MathType@MTEF@5@5@+=feaafiart1ev1aaatCvAUfKttLearuWrP9MDH5MBPbIqV92AaeXatLxBI9gBaebbnrfifHhDYfgasaacH8akY=wiFfYdH8Gipec8Eeeu0xXdbba9frFj0=OqFfea0dXdd9vqai=hGuQ8kuc9pgc9s8qqaq=dirpe0xb9q8qiLsFr0=vr0=vr0dc8meaabaqaciaacaGaaeqabaqabeGadaaakeaacqWGebardaqhaaWcbaGaeGOmaidabaGaey4fIOcaaaaa@2FCB@ to D1∗
 MathType@MTEF@5@5@+=feaafiart1ev1aaatCvAUfKttLearuWrP9MDH5MBPbIqV92AaeXatLxBI9gBaebbnrfifHhDYfgasaacH8akY=wiFfYdH8Gipec8Eeeu0xXdbba9frFj0=OqFfea0dXdd9vqai=hGuQ8kuc9pgc9s8qqaq=dirpe0xb9q8qiLsFr0=vr0=vr0dc8meaabaqaciaacaGaaeqabaqabeGadaaakeaacqWGebardaqhaaWcbaGaeGymaedabaGaey4fIOcaaaaa@2FC9@. This is in contradiction with the assumption that both dose distributions are globally optimum).

In [1], it was conjectured that because all globally degenerate optima share the same modes of solving the fundamental conflicts of the problem between target and normal tissue objectives, the structure of the subspace of high curvature around them is universal. Together with the universality of the subspace of zero curvature, this earlier conjecture would be a consequence of the following, more general formulation: Let D
 MathType@MTEF@5@5@+=feaafiart1ev1aaatCvAUfKttLearuWrP9MDH5MBPbIqV92AaeXatLxBI9gBamrtHrhAL1wy0L2yHvtyaeHbnfgDOvwBHrxAJfwnaebbnrfifHhDYfgasaacH8akY=wiFfYdH8Gipec8Eeeu0xXdbba9frFj0=OqFfea0dXdd9vqai=hGuQ8kuc9pgc9s8qqaq=dirpe0xb9q8qiLsFr0=vr0=vr0dc8meaabaqaciaacaGaaeqabaWaaeGaeaaakeaaimaacqWFdepraaa@3827@* be the set of all globally optimum dose distributions and let Φ* be the associated set of all fluence weight distributions with respect to the basis ℬ
 MathType@MTEF@5@5@+=feaafiart1ev1aaatCvAUfKttLearuWrP9MDH5MBPbIqV92AaeXatLxBI9gBamrtHrhAL1wy0L2yHvtyaeHbnfgDOvwBHrxAJfwnaebbnrfifHhDYfgasaacH8akY=wiFfYdH8Gipec8Eeeu0xXdbba9frFj0=OqFfea0dXdd9vqai=hGuQ8kuc9pgc9s8qqaq=dirpe0xb9q8qiLsFr0=vr0=vr0dc8meaabaqaciaacaGaaeqabaWaaeGaeaaakeaaimaacqWFSeIqaaa@377E@*. Let the set Φ_0 _span the subspace of zero curvature of *F*(D*), D* ∈ D
 MathType@MTEF@5@5@+=feaafiart1ev1aaatCvAUfKttLearuWrP9MDH5MBPbIqV92AaeXatLxBI9gBamrtHrhAL1wy0L2yHvtyaeHbnfgDOvwBHrxAJfwnaebbnrfifHhDYfgasaacH8akY=wiFfYdH8Gipec8Eeeu0xXdbba9frFj0=OqFfea0dXdd9vqai=hGuQ8kuc9pgc9s8qqaq=dirpe0xb9q8qiLsFr0=vr0=vr0dc8meaabaqaciaacaGaaeqabaWaaeGaeaaakeaaimaacqWFdepraaa@3827@*, which is assumed to be locally convex around D
 MathType@MTEF@5@5@+=feaafiart1ev1aaatCvAUfKttLearuWrP9MDH5MBPbIqV92AaeXatLxBI9gBamrtHrhAL1wy0L2yHvtyaeHbnfgDOvwBHrxAJfwnaebbnrfifHhDYfgasaacH8akY=wiFfYdH8Gipec8Eeeu0xXdbba9frFj0=OqFfea0dXdd9vqai=hGuQ8kuc9pgc9s8qqaq=dirpe0xb9q8qiLsFr0=vr0=vr0dc8meaabaqaciaacaGaaeqabaWaaeGaeaaakeaaimaacqWFdepraaa@3827@*. *It is conjectured that all third and higher order derivatives of F with respect to any fluence vector in *Φ_0 _*vanish for all dose distributions in *D
 MathType@MTEF@5@5@+=feaafiart1ev1aaatCvAUfKttLearuWrP9MDH5MBPbIqV92AaeXatLxBI9gBamrtHrhAL1wy0L2yHvtyaeHbnfgDOvwBHrxAJfwnaebbnrfifHhDYfgasaacH8akY=wiFfYdH8Gipec8Eeeu0xXdbba9frFj0=OqFfea0dXdd9vqai=hGuQ8kuc9pgc9s8qqaq=dirpe0xb9q8qiLsFr0=vr0=vr0dc8meaabaqaciaacaGaaeqabaWaaeGaeaaakeaaimaacqWFdepraaa@3827@*. The first and second order derivatives vanish by virtue of the optimality conditions and the above considerations following eq. 7.

A direct consequence of this conjecture is, that the subspace of non-vanishing curvature of *H*_*ij*_(D*) is also an invariant and the matrix *H*_*ij*_(D*) is constant for all dose distributions in D
 MathType@MTEF@5@5@+=feaafiart1ev1aaatCvAUfKttLearuWrP9MDH5MBPbIqV92AaeXatLxBI9gBamrtHrhAL1wy0L2yHvtyaeHbnfgDOvwBHrxAJfwnaebbnrfifHhDYfgasaacH8akY=wiFfYdH8Gipec8Eeeu0xXdbba9frFj0=OqFfea0dXdd9vqai=hGuQ8kuc9pgc9s8qqaq=dirpe0xb9q8qiLsFr0=vr0=vr0dc8meaabaqaciaacaGaaeqabaWaaeGaeaaakeaaimaacqWFdepraaa@3827@*. The parameter space ℱ
 MathType@MTEF@5@5@+=feaafiart1ev1aaatCvAUfKttLearuWrP9MDH5MBPbIqV92AaeXatLxBI9gBamrtHrhAL1wy0L2yHvtyaeHbnfgDOvwBHrxAJfwnaebbnrfifHhDYfgasaacH8akY=wiFfYdH8Gipec8Eeeu0xXdbba9frFj0=OqFfea0dXdd9vqai=hGuQ8kuc9pgc9s8qqaq=dirpe0xb9q8qiLsFr0=vr0=vr0dc8meaabaqaciaacaGaaeqabaWaaeGaeaaakeaaimaacqWFXeIraaa@3787@ becomes a direct product of the space of vanishing curvature ℱ
 MathType@MTEF@5@5@+=feaafiart1ev1aaatCvAUfKttLearuWrP9MDH5MBPbIqV92AaeXatLxBI9gBamrtHrhAL1wy0L2yHvtyaeHbnfgDOvwBHrxAJfwnaebbnrfifHhDYfgasaacH8akY=wiFfYdH8Gipec8Eeeu0xXdbba9frFj0=OqFfea0dXdd9vqai=hGuQ8kuc9pgc9s8qqaq=dirpe0xb9q8qiLsFr0=vr0=vr0dc8meaabaqaciaacaGaaeqabaWaaeGaeaaakeaaimaacqWFXeIraaa@3787@_0 _and the space of positive curvature ℱ
 MathType@MTEF@5@5@+=feaafiart1ev1aaatCvAUfKttLearuWrP9MDH5MBPbIqV92AaeXatLxBI9gBamrtHrhAL1wy0L2yHvtyaeHbnfgDOvwBHrxAJfwnaebbnrfifHhDYfgasaacH8akY=wiFfYdH8Gipec8Eeeu0xXdbba9frFj0=OqFfea0dXdd9vqai=hGuQ8kuc9pgc9s8qqaq=dirpe0xb9q8qiLsFr0=vr0=vr0dc8meaabaqaciaacaGaaeqabaWaaeGaeaaakeaaimaacqWFXeIraaa@3787@_+_. In practice, three obstacles to validating this conjecture for a given example exist. Firstly, the basis set ℬ
 MathType@MTEF@5@5@+=feaafiart1ev1aaatCvAUfKttLearuWrP9MDH5MBPbIqV92AaeXatLxBI9gBamrtHrhAL1wy0L2yHvtyaeHbnfgDOvwBHrxAJfwnaebbnrfifHhDYfgasaacH8akY=wiFfYdH8Gipec8Eeeu0xXdbba9frFj0=OqFfea0dXdd9vqai=hGuQ8kuc9pgc9s8qqaq=dirpe0xb9q8qiLsFr0=vr0=vr0dc8meaabaqaciaacaGaaeqabaWaaeGaeaaakeaaimaacqWFSeIqaaa@377E@* can only be approximated by a finite basis set. Secondly, the computation of the Hessian matrix is extremely time consuming for large basis sets, since it grows quadratically with the number of basis fluence elements. Thirdly, the conjecture cannot be proven numerically because the size of the derivative tensors grows exponentially with the order of the derivative.

In order to visualize the putatively universal curvature properties with a minimum of computational effort, the concept of the *curvature map *is introduced, which avoids these problems. Let D* be a globally optimum dose distribution and let ℬ
 MathType@MTEF@5@5@+=feaafiart1ev1aaatCvAUfKttLearuWrP9MDH5MBPbIqV92AaeXatLxBI9gBamrtHrhAL1wy0L2yHvtyaeHbnfgDOvwBHrxAJfwnaebbnrfifHhDYfgasaacH8akY=wiFfYdH8Gipec8Eeeu0xXdbba9frFj0=OqFfea0dXdd9vqai=hGuQ8kuc9pgc9s8qqaq=dirpe0xb9q8qiLsFr0=vr0=vr0dc8meaabaqaciaacaGaaeqabaWaaeGaeaaakeaaimaacqWFSeIqaaa@377E@_*p *_be any set of arbitrarily chosen *probing fluence elements*, then the *curvature map *(*CM*) is defined as

M(ηi∈ℬp):=∑j=1mDj∗∂2f(D∗)∂Dj2Tjηi.     (9)
 MathType@MTEF@5@5@+=feaafiart1ev1aaatCvAUfKttLearuWrP9MDH5MBPbIqV92AaeXatLxBI9gBamrtHrhAL1wy0L2yHvtyaeHbnfgDOvwBHrxAJfwnaebbnrfifHhDYfgasaacH8akY=wiFfYdH8Gipec8Eeeu0xXdbba9frFj0=OqFfea0dXdd9vqai=hGuQ8kuc9pgc9s8qqaq=dirpe0xb9q8qiLsFr0=vr0=vr0dc8meaabaqaciaacaGaaeqabaWaaeGaeaaakeaacqWGnbqtcqGGOaakiiGacqWF3oaAdaWgaaWcbaGaemyAaKgabeaakiabgIGioJWaaiab+XsicnaaBaaaleaacqWGWbaCaeqaaOGaeiykaKIaeiOoaOJaeyypa0ZaaabCaeaacqWGebardaqhaaWcbaGaemOAaOgabaGaey4fIOcaaaqaaiabdQgaQjabg2da9iabigdaXaqaaiabd2gaTbqdcqGHris5aOWaaSaaaeaacqGHciITdaahaaWcbeqaaiabikdaYaaakiabdAgaMjabcIcaOiabdseaenaaCaaaleqabaGaey4fIOcaaOGaeiykaKcabaGaeyOaIyRaemiraq0aa0baaSqaaiabdQgaQbqaaiabikdaYaaaaaGccqWGubavdaWgaaWcbaGaemOAaOgabeaakiab=D7aOnaaBaaaleaacqWGPbqAaeqaaOGaeiOla4IaaCzcaiaaxMaadaqadaqaaiabiMda5aGaayjkaiaawMcaaaaa@64C3@

The finite set of probing fluence elements is assumed to be a subset of the fictitious basis set ℬ
 MathType@MTEF@5@5@+=feaafiart1ev1aaatCvAUfKttLearuWrP9MDH5MBPbIqV92AaeXatLxBI9gBamrtHrhAL1wy0L2yHvtyaeHbnfgDOvwBHrxAJfwnaebbnrfifHhDYfgasaacH8akY=wiFfYdH8Gipec8Eeeu0xXdbba9frFj0=OqFfea0dXdd9vqai=hGuQ8kuc9pgc9s8qqaq=dirpe0xb9q8qiLsFr0=vr0=vr0dc8meaabaqaciaacaGaaeqabaWaaeGaeaaakeaaimaacqWFSeIqaaa@377E@* and is prompted by practical reasons only. This set can be chosen freely. Notice that due to the (local) convexity of *F*, the CM cannot be negative. If the conjecture holds, the curvature maps of all dose distributions that are degenerate to the global optimum are equivalent.

The globally optimum solution is essentially determined by those volumes in the patient where it is hard to meet target volume or normal tissue objectives. If a probing fluence element *η *passes through these volumes where the dose distribution is a forced compromise, its corresponding CM-value *M*(*η*) will be high. In contrast, if a probing fluence element passes only through volumes where the target and normal tissue objectives can be met, the CM-value will be close to zero.

One advantageous choice of probing fluence elements for beam direction optimisation could be the 'generalized Gamma knife basis set', a set of conical beams of a certain diameter of 15–20 mm say, which impinge on the isocentre from about 5000 directions distributed uniformly across the unit sphere. The curvature map would then show beam directions which pass through or avoid areas of conflicts between dose prescriptions. There are no limits to the choice of ℬ
 MathType@MTEF@5@5@+=feaafiart1ev1aaatCvAUfKttLearuWrP9MDH5MBPbIqV92AaeXatLxBI9gBamrtHrhAL1wy0L2yHvtyaeHbnfgDOvwBHrxAJfwnaebbnrfifHhDYfgasaacH8akY=wiFfYdH8Gipec8Eeeu0xXdbba9frFj0=OqFfea0dXdd9vqai=hGuQ8kuc9pgc9s8qqaq=dirpe0xb9q8qiLsFr0=vr0=vr0dc8meaabaqaciaacaGaaeqabaWaaeGaeaaakeaaimaacqWFSeIqaaa@377E@* other than practicality and visualization.

## Results and Discussion

**Figure 1:**
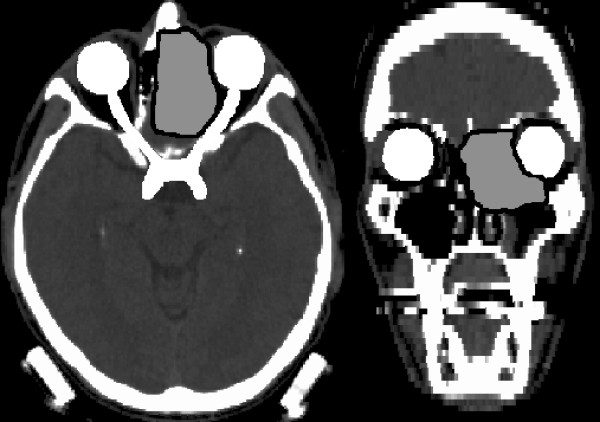
**Geometry of the example case**. Transversal and coronal sections of the example case, a paranasal sinus tumour. Target volume outlined in black/dark grey, optical pathway light grey.

**Figure 2:**
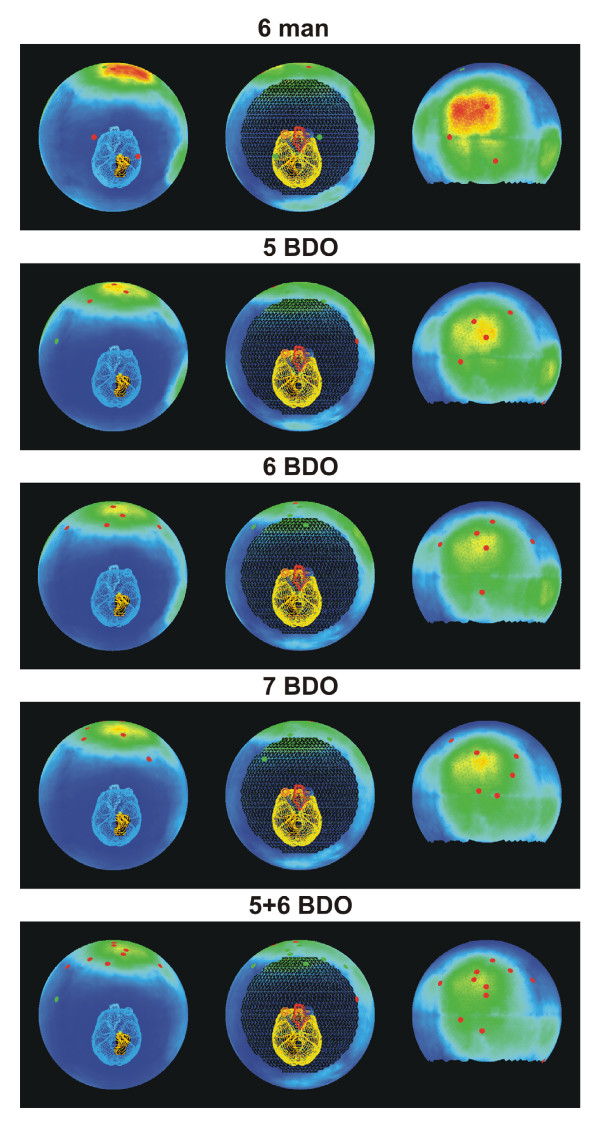
**Curvature Maps for the example case**. Curvature maps of a manually optimised 6 beam configuration, a 5, 6, and 7 beam direction optimised configuration, and a 11 field configuration consisting of the union of the 5 BDO and 6 BDO configurations. The CM values were mapped onto a sphere for 5000 rays which sample all feasible directions of incidence. Left column: cranial view, second column: caudal view, third column: frontal view. The black excision corresponds to beam angles that were excluded due to couch/gantry collisions. Blue sectors correspond to a CM-value of zero, red and yellow regions display zones of conflicts between target objectives and normal tissue constraints. The colour scale is equivalent for all rows. Beam directions are given as red dots.

The example case presented here is a paranasal sinus tumour, see figure 1. The obvious obstacle for achieving the target dose is the proximity of the optical pathway and to a much smaller degree the brain. The setup of the optimisation problem included hard constraints on the maximum equivalent uniform dose (EUD) [3,4] of the organs at risk (optical pathways, brain, brainstem, unspecified tissue), and a hard constraint for the maximum dose to the target. For the target, the objective was to maximize EUD under the given constraints. The sum of these constituent objective functions, together with their appropriate Lagrange multipliers, yields the objective function *F*. With this combination of physical and EUD constraints, the objective function is convex. The Lagrange multipliers are fixed such that the constraints are obeyed for the solution D*, with the consequence that the sole floating figure of merit is the EUD of the target volume. In figure 2, curvature maps for several beam configurations are shown. The CM value is colour coded, starting from zero in blue to the common maximum in red, i.e. all CMs cover the same range of curvatures. Beam directions are indicated as dots on the sphere.

Several observations can be made.

• The rows two through four show CMs of a five, six and seven IMRT beam configuration created by a beam direction optimisation (BDO) algorithm [5]. The first row shows a beam configuration which was chosen manually (6 MAN). The difference between 5 BDO and 6 BDO is somewhat greater than between 6 BDO and 7 BDO, but the general distribution of curvatures is rather constant, with a noticable shrinkage of the total area covered in red and yellow. This impression of increasing proximity to the global optimum is also supported by their final objective function values, which were 97 per cent and 99 per cent respectively of the EUD of the 7 BDO configuration. Notice, that each configuration has no more than one beam in common. The difference between the BDO plans would be greater if they were scaled to their common maximum, rather than the maximum of 6 MAN.

• The first row shows the CM of a manually selected 6 field configuration. The CM differs noticeably from the 7 BDO CM. At the same time, only 94 per cent of the EUD of 7 BDO could be reached. The frontal red region is more extensive in the 6 MAN configuration, which shows that a larger sector of the unit sphere is affected by conflict volumes in the patient. There is one beam direction which originates from a blue region. Blue sectors indicate that directions lie in the subspace of vanishing curvature and thus can be easily substituted. If possible, the BDO algorithm eliminates these directions. In contrast, the human expert picked this direction on the basis of intuition, which was misleading in this case.

• The fifth row shows the CM for the combined 5 BDO and 6 BDO configuration (i.e. an optimized dose distribution of 11 beams). Notice that there is virtually no difference to the 7 BDO configuration. The combined configuration yields the same EUD in the target as 7 BDO. This indicates, that about 7 beams suffice to generate a globally optimum dose distribution for this case, and that a 11 beam arrangement can contain several superfluous beams.

• Notice that in any case, since the objective function in this example is convex, at least the blue areas of the CM of two globally optimum dose distributions have to agree.

Naturally, the basis of probing fluence elements is never complete. In this example, it was chosen such that the full extent of the patient is covered. For more extensive target volumes, different choices may be more appropriate. Notice, that according to the above conjecture, the CM of globally optimum dose distributions should be equivalent for any choice of basis.

If the CM is applied in beam direction optimisation, it provides complementary information to the objective function value. Iterative beam selection has to continue as long as the CM changes between successive configurations. If two rival configurations differ in their CMs, neither of them is truly equivalent to the global optimum. By virtue of the above conjecture, the CM contributes supplemental information in case the beam selection process gets stuck with two rival beam arrangements which produce the same objective function value, yet could be improved further. Once a final configuration was found, beams originating from blue zones are most likely redundant and can be removed from the configuration. Notice, that the CM cannot be used to guide the search for additional beam directions at intermediate stages: since it is subject to significant change while the beam configuration evolves, the indication of redundant beams is not stable. The first derivative with respect to the weight of a probing fluence element may offer more information for this purpose.

## Conclusion

It is conjectured for the IMRT optimisation problem, that in the proximity of the global optimum, the second and all higher order derivatives vanish in a subspace of sizeable dimension. As a consequence, the curvature of the objective function for any globally optimum dose distribution is equivalent. In order to verify this property, the tool of a curvature map was introduced which relies on a freely choosable set of probing fluence elements. This allows to compare the curvature properties of dose distribution which have no beams in common. These considerations about curvature are intended to highlight the special properties of the IMRT optimisation problem, which may further its understanding or be used to advantage in algorithm design.

## Authors' contributions

MA and GM developed the maths and prepared the manuscript. GM coded the software tools and prepared the example.
